# Commentary: Lack of functional specialization of neurons in the mouse primary visual cortex that have expressed calretinin

**DOI:** 10.3389/fnins.2016.00230

**Published:** 2016-05-24

**Authors:** Filip Barinka

**Affiliations:** ^1^Department of Neurology, University of RegensburgRegensburg, Germany; ^2^Department of Psychiatry, University of RegensburgRegensburg, Germany

**Keywords:** calretinin, visual cortex, interneurons, visual response properties, orientation tuning, spatial frequency

The intracellular protein calretinin (CR) is frequently used as a marker of specific (albeit heterogeneous) cortical interneuronal population (see Cauli et al., 2014; Schwaller, 2014 for recent reviews). A large body of data on connectivity of calretinin expressing (CR+) neocortical interneurons in various areas and species has been collected in the last two decades. However, remarkably little is yet known about their reactions to physiological stimuli during information processing in neocortical networks. Furthermore, we still lack precise data about the physiological roles of calretinin in neocortical neurons and we also do not know whether the presence of calretinin protein in these neurons is directly coupled to any special physiological feature of these cells. In other words, are there any common functional attributes, shared (exclusively?) by (all?) CR+ neocortical neurons?

Alexander Heimel from the Netherlands Institute for Neuroscience with his research group decided to challenge this topic by characterizing the physiological response properties of CR+ interneurons in the primary visual cortex of the mouse *in vivo* (Camillo et al., 2014). They intended to figure out, whether these cells differ in their receptive field properties from the general neuronal population in this area.

With this intention, a very elegant study design was elaborated, combining various methods: the use of genetically modified mouse lines, two-photon calcium imaging, immunohistochemistry as well as in situ hybridization [the last one retrieved as “ready-made” *in situ* hybridization images from the Allen Mouse Brain Atlas (Lein et al., 2007; Oh et al., 2014)].

Firstly, two genetically engineered mouse lines were crossed: a Cre-dependent reporter mouse line expressing the red fluorescent protein tdTomato (tdTom) with a CR-ires-Cre mouse line, which expresses the enzyme Cre-recombinase in a fashion similar to endogenous CR expression. In the offspring from this cross, the cellular expression of the tdTom label was indirectly activated by the expression of CR. In these animals, the orientation selectivity, size tuning, and temporal and spatial frequency tuning of tdTom+ cells were estimated *in vivo* by measuring the intracellular calcium changes via two-photon calcium imaging using the genetically encoded calcium indicator GCaMP6s, while showing standardized visual stimuli.

After comparing the results with those acquired from the general tdTom negative neuronal population, the authors concluded that in none of the studied visual response properties (VRPs) the tdTom+ population is significantly different from the tdTom– population.

On the first sight, it would suggest that the CR+ (inter)neurons do not differ in their VRPs from the general neuronal population, composed of the pyramidal neurons and other (CR negative) interneuronal types.

However, when the expression of tdTom label was directly compared with the expression of CR (via immunohistochemistry and in situ hybridization), it could be shown that only 20% of tdTom+ cells expressed also calretinin. By contrast, 96% of CR+ cells expressed also tdTom. This showed that actually many of tdTom+ neurons expressed CR only transiently during their development. And indeed, the fact that 60% of tdTom+ cells also expressed SatB2 (marker of pyramidal neurons) underlines the conclusion that the majority of tdTom+ cells are pyramidal neurons which expressed CR transiently during their development. A further comparison with other interneuronal markers (parvalbumin—PV, somatostatin—SOM) showed a low colocalization between CR and SOM and no colocalization between CR and PV. Hence, the exclusive expression of CR or PV (but never both of them) in adult cortical neurons well-known across all studied species including mouse (Xu et al., 2010) seems to be also valid during the ontogeny of the cortical neurons in the mouse visual cortex. This is an interesting and not automatically obvious point, as well as a transient co-expression of PV and CB (Hendrickson et al., 1991; Hogan and Berman, 1993; Yan et al., 1995) and even transient neuronal co-expression of PV and CR (Yan et al., 1995) has been reported in other species. The work of Daniela Camillo and colleagues so accentuates the presence of significant interspecies differences in cellular composition of cortical networks. It also makes us aware how little information we still have about the physiological functions of protein calretinin in neocortical neurons both in ontogenesis and in mature cortex. To mention only one more of question opened when analyzing the results of Camillo et al. (2014), it remains to be elucidated why only a portion of pyramidal neurons transiently express calretinin and how do they differ from the remaining population. (For further reading on functions of calretinin and on the developmental pattern of CR expression in human cortex see also other papers of the same Frontiers Research Topic collection (Gonzalez-Gomez and Meyer, 2014; Radonjic et al., 2014; Schwaller, 2014).

Finally, coming back to the VRPs of tdTom+ cells, it has to be acknowledged that the heterogeneity of the tdTom+ population (transitory CR+ pyramidal neurons and adult CR+ interneurons) complicates the interpretation of the results with regard to the VRPs of these cells. The study of Camillo et al. shows that the neurons with a common “CR history” do not differ in their VRPs from the general neuronal population. In earlier studies, significant differences of some of the VRPs between excitatory neurons and GABAergic interneurons have been documented in mouse visual cortex. In some of those studies, a discrimination between individual types of inhibitory neurons was not (Sohya et al., 2007) or only partially (fast-spiking vs. regular-spiking neurons) (Liu et al., 2009) possible. But, in the work of Kerlin et al. (2010), the PV+, SOM+, and VIP+ interneurons could be discriminated and showed to possess similar VRPs that differ from those of pyramidal neurons. As there is a significant (but not complete) overlap of expression of CR and VIP, a difference of VRPs between CR+ interneurons and pyramidal excitatory neurons can be presumed. However, the question whether the CR+ interneurons in the adult visual cortex really differ in their VRPs from other interneuronal classes and from excitatory neurons remains unanswered. A modification of study design (see Camillo et al., 2014, for suggestions) will be necessary for answering this question. Nonetheless, the study of Daniela Camillo and colleagues shows a feasibility of such an approach and, as mentioned above, opens many interesting questions concerning calretinin and CR+ neocortical interneurons which also need to be addressed in the future.

## Author contributions

The author confirms being the sole contributor of this work and approved it for publication.

### Conflict of interest statement

The author declares that the research was conducted in the absence of any commercial or financial relationships that could be construed as a potential conflict of interest.
